# Validation of the Filovirus Plaque Assay for Use in Preclinical Studies

**DOI:** 10.3390/v8040113

**Published:** 2016-04-21

**Authors:** Amy C. Shurtleff, Holly A. Bloomfield, Shannon Mort, Steven A. Orr, Brian Audet, Thomas Whitaker, Michelle J. Richards, Sina Bavari

**Affiliations:** 1 Molecular and Translational Sciences Division, United States Army Medical Research Institute of Infectious Diseases, 1425 Porter Street, Frederick, MD 21702, USA; sina.bavari.civ@mail.mil; 2 Center for Aerobiological Studies, United States Army Medical Research Institute of Infectious Diseases, 1425 Porter Street, Frederick, MD 21702, USA; holly.a.bloomfield.civ@mail.mil; 3 Nonclinical Development Division, United States Army Medical Research Institute of Infectious Diseases, 1425 Porter Street, Frederick, MD 21702, USA; shannon.m.mort.ctr@mail.mil (S.M.); sorr@southernresearch.org (S.A.O); 4 Cell Culture Laboratory, Virology Division, United States Army Medical Research Institute of Infectious Diseases, 1425 Porter Street, Frederick, MD 21702, USA; brian.e.audet.civ@mail.mil (B.A.); thomas.j.whitaker26.ctr@mail.mil (T.W.); michelle.j.richards.civ@mail.mil (M.J.R.)

**Keywords:** plaque assay, filovirus, Ebola, ebolavirus, Marburgvirus, Marburg virus, Vero E6 cells, GLP compliant, validation, animal rule

## Abstract

A plaque assay for quantitating filoviruses in virus stocks, prepared viral challenge inocula and samples from research animals has recently been fully characterized and standardized for use across multiple institutions performing Biosafety Level 4 (BSL-4) studies. After standardization studies were completed, Good Laboratory Practices (GLP)-compliant plaque assay method validation studies to demonstrate suitability for reliable and reproducible measurement of the Marburg Virus Angola (MARV) variant and Ebola Virus Kikwit (EBOV) variant commenced at the United States Army Medical Research Institute of Infectious Diseases (USAMRIID). The validation parameters tested included accuracy, precision, linearity, robustness, stability of the virus stocks and system suitability. The MARV and EBOV assays were confirmed to be accurate to ±0.5 log_10_ PFU/mL. Repeatability precision, intermediate precision and reproducibility precision were sufficient to return viral titers with a coefficient of variation (%CV) of ≤30%, deemed acceptable variation for a cell-based bioassay. Intraclass correlation statistical techniques for the evaluation of the assay’s precision when the same plaques were quantitated by two analysts returned values passing the acceptance criteria, indicating high agreement between analysts. The assay was shown to be accurate and specific when run on Nonhuman Primates (NHP) serum and plasma samples diluted in plaque assay medium, with negligible matrix effects. Virus stocks demonstrated stability for freeze-thaw cycles typical of normal usage during assay retests. The results demonstrated that the EBOV and MARV plaque assays are accurate, precise and robust for filovirus titration in samples associated with the performance of GLP animal model studies.

## 1. Introduction

Filoviruses are zoonotic viruses belonging to the virus family *Filoviridae* and can cause severe hemorrhagic fever in humans and Nonhuman Primates (NHP), with high case fatality rates. These viruses, specifically Ebola (EBOV) and Marburg (MARV) viruses, are some of the most lethal viruses known to man. Filoviruses can be detected and quantified using a variety of basic or sophisticated virological methods. These methods include, but are not limited to plaque assays, reverse-transcription polymerase chain reaction (RT-PCR), deep sequencing, transmission electron microscopy (TEM), the 50% tissue culture infectious dose assay (TCID_50_) and ViroCyt^®^ flow-based methods, each with their own limitations for detection and quantitation of virus genomic material, viral proteins or intact infectious particles [1,2,3,4].

The viral plaque assay is a widely-used technique for virus isolation and purification and the quantitation of infectious viral particles within a sample [5]. The basis of the technique is to measure the ability of a plaque-forming unit (PFU) of virus to form a “plaque” on a confluent monolayer culture of adherent cells. A plaque results from the infection of a cell by a plaque-forming unit followed by the replication of that virus and, eventually, the death of the cell. From that cell, the newly-replicated virus particles infect and kill surrounding cells. The culture is then stained with a dye, discerning the cells in the plaque from the healthy surrounding monolayer due to the properties of the stain. The stain can be a vital dye, a protein stain or a virus-specific stain, such as a coupled antibody. The vital dye neutral red plaque assay has been in use at USAMRIID for many years to enumerate EBOV and MARV, in stock solutions and prepared challenge material. The assay is also frequently used to measure filovirus samples from infected NHP in support of animal model development and countermeasures testing projects at USAMRIID. The reliable and accurate measurement of virus in stock solutions, challenge material and NHP blood samples is an essential activity supporting animal model studies and regulated studies for the advanced development of Medical Countermeasures (MCM) under Good Laboratory Practice-compliant (GLP) research conditions [6]. Due to its use in support of regulated studies, this plaque assay must be validated for its intended use [7].

The purpose of these EBOV and MARV validation studies was to demonstrate that the plaque assay was suitable for reliable and reproducible measurement of infectious EBOV and MARV in virus stock solutions, which are routinely diluted to prepare challenge solutions. This study did not include validation of the quantitation of EBOV virus in test samples, such as serum and plasma from species of infected NHP; this was only completed for MARV. The study tested cell passages of the Vero E6 cells that are currently in use at USAMRIID. Based on the nature and intended application of the plaque assay, the EBOV and MARV plaque assays were validated for the following parameters: accuracy, precision, linearity, limit of detection (LOD), lower limit of quantification (LLOQ), robustness, stability and system suitability. For precision studies, two analysts performed the neutral red plaque assay in parallel on two separate days and collected their own data, as well as re-counted each other’s plates. Precision studies resulted in a very high degree of similarity in the data generated by two analysts.

## 2. Materials and Methods

### 2.1. Virus

A USAMRIID cell culture stock of Ebola virus/H. sapiens-tc/COD/1995/Kikwit-9510621 (EBOV) originated from an Ebola Virus Disease (EVD) outbreak in Kikwit, Zaire, in 1995 and was passaged twice at the Centers for Disease Control and Prevention (Atlanta, GA, USA) and twice in Vero E6 cells at USAMRIID for a total of 4 passages. This stock was named “R4370”, [8], and it was used to prepare the Positive Control (PC) and high, medium and low titer Quality Control samples (QC1, QC2 and QC3, respectively), which were used exclusively as the test samples in these studies. The nominal titers of these stocks were determined by plaque assay [9] at the time of stock generation and are presented in Table 1.

A USAMRIID cell culture stock of Marburg virus/H. sapiens-tc/AGO/2005/Angola-200501379/2005 (MARV) originated from a Marburg Virus Disease (MVD) outbreak in Angola in 2005 and was passaged 4 times in Vero E6 cells. This stock was named AIMS (Agent Inventory Management System) number 17214, and it was used to produce the MARV PC, QC1, QC2 and QC3 samples used on the MARV validation study (Table 2). The plaque assay Negative Control (NC) was always the plaque assay medium diluent (1 × MEM with 5% FBS) into which the PC and QC samples were diluted for each experiment. Plated NC never resulted in any plaques for any experimental run.

### 2.2. Cells

The specific Vero E6 cells cultured and plated for the plaque assay (C1008) were obtained from BEI Resources at passage Number 24 (Manassas, VA, USA). BEI Vero E6 cells are Catalog Number NR-596, Lots 3956812 and 3956593. Passage numbers presented in the Results Section denote cumulative passage numbers upon receipt plus subsequent passages at USAMRIID. Cells were cultured under GLP conditions, where all media, supplements and procedures for passaging and culturing cells were performed and recorded by GLP-trained technicians in the USAMRIID Cell Culture Laboratory. Following cell culturing, counting and plating Standard Operating Procedures (SOPs) at USAMRIID, cells were counted by use of either a Cedex HiRes Multi Sampler System (Roche, Indianapolis, IN, USA), a NucleoCounter NC200 (Chemometec, Davis, CA, USA) or a hemocytometer. Cells were plated in 6-well tissue culture dishes (Catalog No. 3506, CoStar, Corning Inc. Corning, NY, USA) or 100-mm tissue culture dishes (Catalog No. 430293, Corning Inc., Corning, NY, USA) at 700,000 ± 25,000 cells/well of a 6-well plate or 4 × 10^6^ ± 150,000 cells/dish in 10 mL of medium for the larger dishes.

### 2.3. Reagents, Cell Culture Media, Supplements and Stains

Filovirus negative, normal cynomolgus macaque and rhesus macaque serum and plasma (K_3_EDTA) were purchased from Bioreclamation (Westbury, NY, USA). Reagents for culturing of cells: 1 × MEM (Cat. No. 10010249, Cellgro, Manassas, VA, USA) in plates, supplemented with 5% to 10% FBS (*v/v*) (Cat. No. 16000-044, Gibco, Life Technologies, Grand Island, NY, USA) and 5 mM L-glutamine (Cat. No. SH330034.01, Thermo Scientific HyClone, GE Healthcare, Logan, UT, USA). Reagents for plaque assay: 2 × _EBME (Eagle’s Basal Medium, labeled as BME, Cat. No. A15950DK, Gibco, Life Technologies, Grand Island, NY, USA), FBS (Fetal bovine serum, Cat. No. SH30071.03 or SH30071.02, Thermo Scientific HyClone, GE Healthcare, Logan, UT, USA), penicillin-streptomycin (Cat. No. 30-022-CI, Cellgro, Manassas, VA, USA), SeaKem ME Agarose (Cat. No. 50014, Lonza, Rockland, ME, USA) and neutral red from Gibco (Cat. No. 02-0066DG, Gibco, Life Technologies, Grand Island, NY, USA).

### 2.4. GLP Compliance

These filovirus plaque assay validation studies were conducted at USAMRIID in compliance with the U.S. Food and Drug Administration Good Laboratory Practice (GLP) Regulations, 21 CFR Part 58. Each validation was conducted according to a GLP study protocol, with any GLP exceptions, SOP or protocol deviations identified and listed in the protocol and/or reports. The studies were inspected and audited by Quality Assurance personnel at BSL-2 and BSL-4 critical performance phases. Study protocols, all collected data and final reports were audited and archived in compliance with GLP regulations.

### 2.5. Filovirus Plaque Assay

The assay was performed in accordance with published methods [9]. Cells were cultured and plated as described in Section 2.2. Cells were plated in 6-well plates 24 to 72 hours prior to use in the assay, as required based on experimental design, and plates were carried into the BSL-4 lab at the time of use. Agarose was prepared at a concentration of 1.0 g agarose per 100 mL distilled water and autoclaved for sterility. Prepared sterilized agarose was re-melted by microwave at BSL-4 prior to each use. Quality control (QC) and positive control (PC) samples were diluted in 1 × MEM, supplemented with 5% FBS (*v/v*), in a 10-fold fashion according to the experimental design. The culture medium in the 6-well plate was removed by swift decanting. Virus inocula at various dilutions were added to duplicate wells of 6-well plates in volumes of 100 or 200 μL, or 1-mL volumes into 100-mm culture dishes. Inoculum fluid was distributed by gentle manual rocking. The plates were incubated at 37 °C for 1 h ± 10 min, rocking every 15 ± 5 min. A primary liquid overlay containing a 1:1 ratio of 2 × EBME medium (supplemented with 10% FBS, 400 IU penicillin, 400 µg/mL streptomycin) equilibrated to 37–42 °C and 1% melted agarose equilibrated to 60 °C degrees was overlaid in 2-mL aliquots onto the inoculated monolayer and allowed to solidify. The inoculum was not removed from the wells prior to the addition of overlay. Plates were incubated for approximately 7 days. At Day 7, they were stained with 2 mL of secondary overlay, which consisted of a 1:1 volume ratio of 2 × EBME plus supplements: 1% agarose and the combined volumes were supplemented with 4% neutral red. Plaques were counted and recorded 24–48 h post-staining. All work with filoviruses was carried out under maximum containment in the BSL-4 laboratories at USAMRIID.

### 2.6. Plaque Counting and Titer Calculation

Plates were inverted on a light box, and plaques were counted by hand with the aid of a marker. For precision experiments, plates were counted once by the performing analyst, then ink marks were gently removed by an ethanol wipe. The plates were then recounted by a second analyst. For experiments requiring recounting on the following day, ink marks were removed, and the plates were placed back into the incubator until the next counting. The SOP method dictated that plaque numbers between 10 and 150 would be counted and recorded and used in calculations to determine quantitative titer measurements. Plaque numbers below 10 were counted and recorded for LOD analyses [5,10]. If there were wells at two different serial dilutions that produced counts within this range, then the counts from wells with the more concentrated dilution were chosen for calculating titer. Counts from replicate wells were averaged, and the average was multiplied by the dilution factor of the inoculum, which produced that number, and the volume of inoculum plated to calculate the plaque forming units (PFU) per mL of the original stock virus preparation. The calculation is: the average value of plaques in replicate wells × dilution factor ÷ virus inoculum volume (in mL, e.g., 0.1 mL) = titer in PFU/mL.

### 2.7. Variables under Test for the Determination of Assay Robustness

Variables, such as passage age of cells, amount of time (in days) cells can be seeded in plates before use, freeze-thaw stability of virus samples in cell culture media, nonhuman primate serum and plasma samples (for MARV only), amount of virus inoculum volume and tissue culture vessel and optimal days upon which to stain and count plaques, were all assessed as part of assay robustness in the validation experiments. Specific details of the experimental design evaluating robustness are presented with the results for that parameter. As experiments progressed over time, the system suitability was determined by analyzing the “stability” of the cells or the effect of cell age (passage number) on plaque number and quality and compared across all experiments, as appropriate.

### 2.8. Statistical Tests

Raw data values were analyzed and described with basic descriptive statistics for presentation as the mean ± standard deviation. Comparisons of sample groups were done assuming equal variance using a two-tailed Student’s *t*-test in Microsoft Excel or GraphPad Prism software [11]. Coefficients of variation (%CV) were calculated and are presented, where relevant [10,12,13].

## 3. Results

### 3.1. Confirmation of Accuracy

The true concentration of virus titer can be measured by an assay that evaluates the amount of live, replicating virus in a sample. To date, the plaque assay is the most reliable method that indicates the true titer, in plaque-forming units (PFU) per mL of sample. The validation protocol established the test-specific acceptance criteria for accuracy as: eight out of at least 10, but up to 12 observed titers for QC1, QC2 and QC3 must be within ± 0.5 log_10_ of their nominal titers, and the Positive Control (PC) sample must be measured within ± 0.5 log_10_ of its nominal titer for the assay to pass the performance criteria [14]. The designated accuracy experiments for MARV and EBOV tested this explicitly. For the accuracy tests performed with each virus, measurements of average titers, %CV and % difference from nominal titer were made and are presented in Table 1 and Table 2.

The % difference of the observed titer from the nominal titer is an important measurement when considering accuracy. The average observed value will be somewhat different from the nominal titer, but it is important to establish an acceptable % difference based on how the assay is known to perform and its natural variability. The titers for MARV and EBOV QC1, QC2 and QC3 are all required to fall within ± 0.5 log_10_ of their nominal titers, and that range provides the values from which the acceptable % differences can be calculated. For example: if the low end of the acceptable titer range is 1.018 × 10^6^ PFU/mL and the nominal titer is 3.52 × 10^6^ PFU/mL, then the % difference is calculated as follows:

$$\frac{1.018\;\times 10^{6}-3.52\;\times \;10^{6}}{3.52\;\times \;10^{6}}\;\times 100\%=\;-71.1\%$$

For this assay run, the EBOV PC value was 6.60 × 10^4^ PFU/mL, which falls in the required PC nominal titer range of 3.45 × 10^4^ (−68.4%) and 3.45 × 10^5^ PFU/mL (+216%) to pass.

For this assay run, MARV PC value was 4.5 × 10^6^ PFU/mL, which falls in the required PC nominal titer range of 1.096 × 10^6^ (−68.7%) to 1.096 × 10^7^ PFU/mL (+218%) to pass.

Most of the other tests on the validation protocol were designed similarly to the accuracy test, with repeated testing of QC1, QC2 and QC3 and the comparison of those results against the nominal titers for EBOV and MARV QCs. The EBOV validation had nine additional tests with this same experimental design, enabling cross-comparison, while the MARV validation had another eight tests. The passage of the accuracy criteria across those tests is presented in Table 3.

The largest observed percentage difference from the nominal titer was seen for MARV Precision 1 QC3 at 175.5%, equating to a value of 4.95 × 10^4^ PFU/mL. This titer is +0.15 log_10_ above the nominal titer. The lowest observed value compared to the nominal titer was that observed for EBOV Stability QC1, which was −54.3%, equating to a value of 5.35 × 10^5^ PFU/mL. This titer is −0.34 log_10_ below the nominal titer. Based on these real tests, the acceptance criterion of ±0.5 log_10_ of the nominal titer is reasonable. Over time and through the use of the assay, it may be possible to reduce the acceptance criterion to a smaller window, but at this time, the criterion of ±0.5 log_10_ is useful to describe the accuracy limits of the assay.

### 3.2. Confirmation of Precision

**Figure 1:**
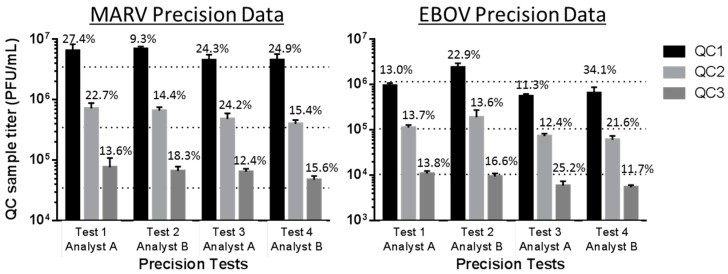
The MARV and EBOV validated plaque assays demonstrate repeatability and intermediate precision. The shaded bars represent average measured titers for QC1, QC2 or QC3 for MARV or EBOV, measured by Analyst A or B in their test performance iteration. Tests 1 and 2 were performed on the same day, but independently by each analyst. Tests 3 and 4 were similarly performed, but on a different day. The three horizontal dotted lines plot the nominal titers of MARV and EBOV QC1, QC2 and QC3. Those nominal titers are presented in Table 1 and Table 2. The %CVs for the average observed QC titers are shown above the relevant bars, and the values were assigned a cutoff for acceptance of ≤30% for this cell-based biological assay based on historical performance and the analysis of system suitability [13,14].

For the precision experiments, QC1, QC2 and QC3 were analyzed four times, once on each of two different days by two analysts per day, working independently. The goal of these experiments was to evaluate the performance of the assay on different days when two independent assays were run in parallel by two separate analysts. The results of these four tests were evaluated for precision, defined as the closeness of individual test results when the procedure is applied repeatedly to multiple aliquots of a single homogeneous sample. The precision of a method is usually expressed as SD or %CV. Precision was considered for: (1) repeatability precision, which indicates how precise the test results are under ideal conditions (same sample, analyst and day); and (2) intermediate precision, which expresses within-laboratory variations, such as different days, different analysts, different instruments or cells from different passage numbers. The acceptance criterion for precision in this validation was defined as a measured %CV of ≤30% for each QC set measured in two paired experiments performed by two analysts, based on historical performance and suitability of the assay for its intended use [10,13,14]. Figure 1 demonstrates the similarities in MARV and EBOV QC set titers measured by Analysts A and B across four tests. The CV measurements are indicated directly above the bars in the graphs. One instance of %CV greater than 30% was observed for the EBOV Test 4 Analyst B QC1 sample (34.1%); however, those Test 4 data were deemed valid because at the time of this assay; the other precision tests and the accuracy tests had already been performed on this validation, and the %CV values all passed the ≤30% criterion. The time, resources and biosafety risk (BSL-4) required to repeat EBOV Test 4 and its companion EBOV Test 3 for the collection of repeated QC data were taken into consideration for the reanalysis decision. Subsequently, out of six more assays performed throughout the EBOV validation, it was found that all other QC datasets passed the %CV criterion.

The analysis of dually-counted data, recorded on separate data sheets, was included in the validation to assess the consistency of plaque counting between two analysts. The precision of plaque counting performed by two technicians was determined by measuring the % variability of mean plaque counts in 24 wells when counted by both analysts on the same set of 12 pairs of plates (two duplicate wells per plate). The % variability was calculated as follows:

$$Variability\;\left(\%\right)=\frac{mean\;plaque\;number\;Analyst\;A\;-mean\;plaque\;number\;Analyst\;B}{\left(\left(mean\;plaque\;number\;Analyst\;A\;+mean\;plaque\;number\;Analyst\;B\right)\;÷2\right)}\times 100$$

The precision test was designed such that analysts counted plaques from their own experiments and then traded plates and independently counted each other’s plates. The sets of QC1, QC2 and QC3 plaque counts recorded by each analyst (*n* = 24 per QC set/analyst) were averaged, and the data were analyzed for % variability between analysts of the overall plaque numbers per QC sample per test. The absolute % variability was required to be less than 30% to pass this acceptance criterion, a cutoff chosen to reflect the ideal of falling within ±0.5 log_10_ of the nominal titer [10,13]. The data demonstrated there were no significant effect on the tallied plaque numbers when the EBOV, and MARV plaques were counted by different analysts (Table 4).

**Figure 2:**
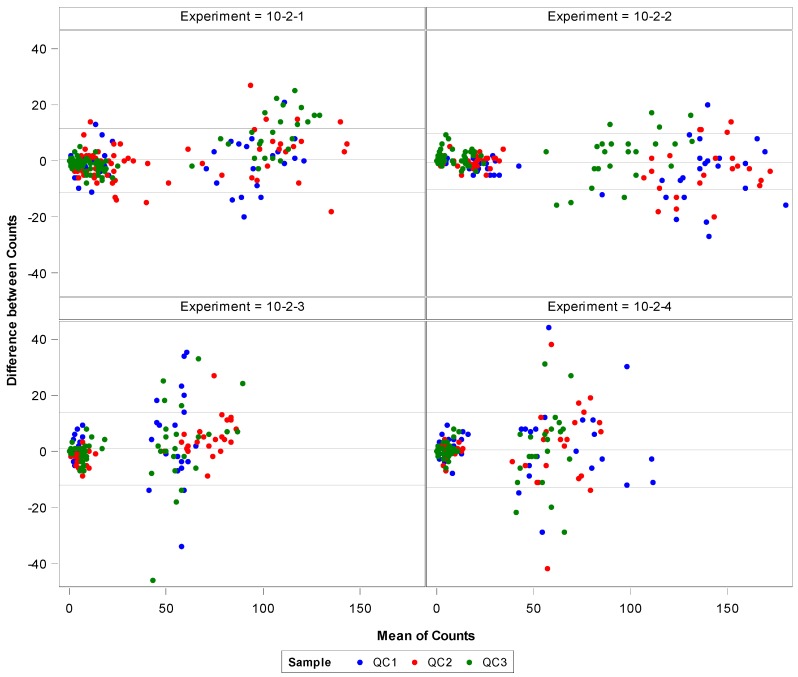
Bland–Altman analysis demonstrates a visual representation of the agreement between plaque numbers counted by two individual analysts.

The Bland–Altman plot (Figure 2) below is a visual representation of the agreement of the EBOV plaque counts made by the two analysts for each precision experiment. The difference between counts of each well are plotted on the *y*-axis, while the mean of the two counts for each well are plotted on the *x*-axis. The central reference line in each plot is the mean of the differences between counts; while the two other reference lines represent two standard deviations of the differences above and below the mean. It appears that the fewer the plaques counted, the closer the data fell to that central reference line: therefore, it is possible that when there are fewer plaques to count, there is less variation between two analysts. The Bland–Altman analysis was also completed for the MARV data set (see Figure S1), and the results were very similar.

In addition, an Intra-Class Correlation (ICC) analysis was performed for evaluating the precision of the assays for EBOV and MARV (Table 5). The ICC technique operates on data organized into test groups. It assesses the consistency or conformity of measurements made by multiple observers measuring the same quantity [15]. ICC was used to analyze the results when variation (e.g., multiple analysts) was introduced to a set for the same samples. Evaluation of the calculated ICC values passed the test-specific acceptance criterion of at least 0.8 for the lower 95% confidence intervals (CI), indicating that plaque count data from two different analysts for the same sets of plates are in high agreement, and there is very little variation between analysts when plaques are counted. Inherently, there must be clear plaques of high quality that are recognizable by a trained analyst. When trained individuals were presented with high quality plaques, both analysts generated similar plaque counts and calculated titer data. Throughout the performance of the validated assay, it will be possible to have multiple users perform the assay for various data collection needs, and the data will be comparable because the assay has repeatability and intermediate precision. ICC coefficients and 95% CI were calculated; demonstrating how strongly the EBOV and MARV plaque count results resemble each other when counted by two analysts in the precision tests (Table 5).

### 3.3. Confirmation of Linearity

The measured average titers of EBOV and MARV QC1, QC2 and QC3 (*n* = 12, from the accuracy dataset; Table 1 and Table 2) were proportional to their respective nominal high, medium and low titers, which demonstrated the linearity of the assay. For the EBOV validation studies, the slope and *R*^2^ values were calculated at 0.52 and 0.8275, respectively. The *R*^2^ value had to be >0.5 to pass the acceptance criterion for linearity [14]. Similarly, for MARV, the slope and *R*^2^ values were calculated at 0.81 and 0.8229, respectively, supporting that both validated plaque assays demonstrated linearity in sample measurement.

### 3.4. Robustness in the Assay

#### 3.4.1. Stability

**Figure 3:**
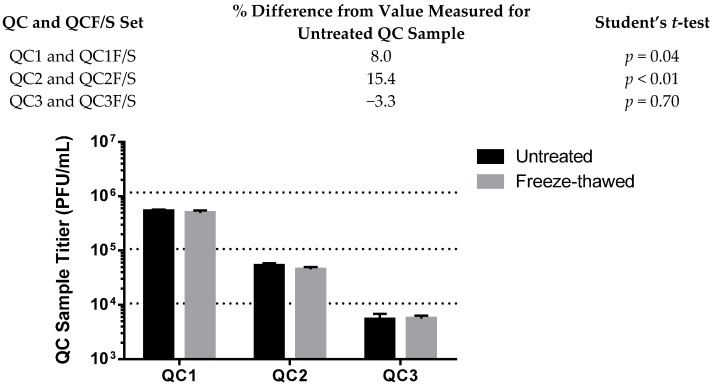
Freeze-thaw treated (designated as F/S) and untreated quality control QC samples 1-3 have very similar measured titers in the EBOV plaque assay.

**Figure 4:**
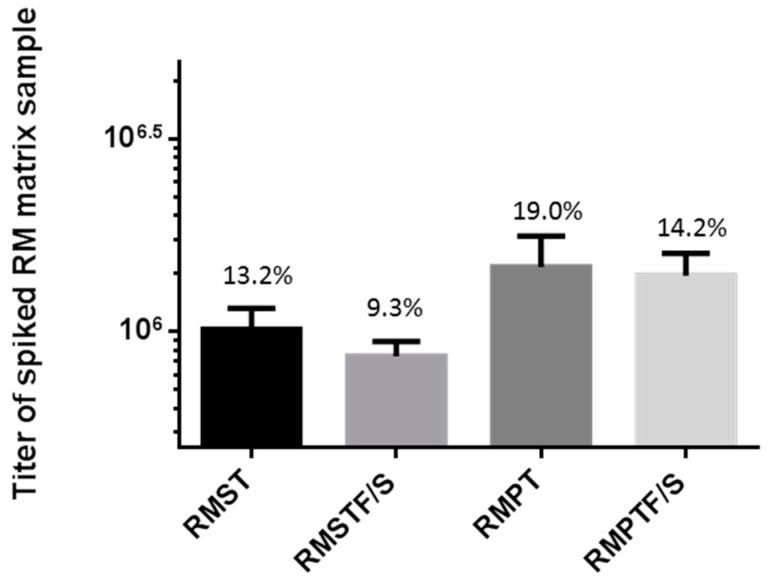
MARV-spiked rhesus macaque serum and plasma can be titered after freeze-thaw cycles. Rhesus Macaque Serum (RMST) and Plasma (RMPT) were subjected to three freeze-thaw cycles of being held thawed at ambient temperature for at least 30 min and refrozen at −60 °C or colder for no less than 20 h, on three consecutive days. Samples were titered in the plaque assay after the fourth thaw. There was little variability in each average titer, and a Student’s *t*-test found a statistically-significant difference (*p* < 0.01) between RMST and RMSTF/S sample sets, yet the titers are acceptably similar; therefore, the difference is not biologically significant.

The stability of the virus stock after multiple freeze-thaw cycles was tested to determine if the virus samples and positive controls were stable during common manipulations. Repeat measurement of samples is sometimes required during the data collection process, especially if an assay fails or other factors beyond a researcher’s control necessitate a repeat performance. Published studies evaluating the environmental stability of filoviruses in conditions of light, heat or humidity are available [16,17,18], but only one reference was found citing the integrity of viruses after freezing and thawing [19]. Although our laboratory has observed the stability of filovirus stocks and samples upon repeated freeze thaw actions, the degree to which titers change after freeze thaw cycles is unknown. Regulatory guidance for approaching reagent stability recommends the evaluation of reasonable laboratory handling conditions [7] and proposes evaluation at a minimum of three freeze-thaw cycles. Three cycles allow for an initial measurement to fail and up to two more repeat measurements, if necessary. For the EBOV validation, only virus diluted in cell culture medium was tested for freeze-thaw cycle stability (Figure 3). For MARV validation studies, cell culture medium, NHP serum and NHP plasma were all matrices tested for effects on virus stability after three freeze-thaw cycles (Figure 4). These matrices were spiked with virus to a titer consistent with QC2. For the EBOV studies, a very similar titer was measured for samples that had been continuously held frozen and those that had been through three cycles of freeze-thawing. The thawing procedure was for no less than 30 min at ambient temperature, which was adequate to ensure complete thawing of the sample, and the refreezing was for no less than 20 h at −60 °C or lower, on three consecutive days (denoted as F/S in Figure 4).

The validation protocol acceptance criterion for stability was that the F/S sample had to return a titer no more than 30% different from the value measured for the non-freeze-thawed sample to demonstrate that freeze-thawing had no detrimental effect on the sample. Consistently, less than a 30% difference was observed for the EBOV and MARV cell culture samples, where the widest range of differences from the untreated samples was 15.4%, and 12.4%, for EBOV QC2 and the MARV QC1, respectively (MARV; data shown in Figure S2). Statistical comparison by Student’s *t*-test of the treated *versus* untreated samples demonstrated statistical significance between the two datasets (*p* = 0.04 for QC1 and *p* < 0.01 for QC2), even though the titers measured for the two groups were very close, thus indicating very low variability in titers measured for each sample group (see also the error bars in Figure 3). The statistical significance in this case did not present a meaningful biological difference in the titers of the treated samples. The practice of using a cell culture sample that has up to three freeze-thaw manipulations is acceptable for the EBOV and MARV validated assays.

The stability of MARV-spiked cynomolgus macaque (CM), or rhesus macaque (RM) serum (ST), or plasma (PT) samples (designated as CMST/CMPT or RMST/RMPT) was tested using virus at a mid-level concentration (the concentration of QC2). To pass the acceptance criterion for this comparison assay in the validation, the titers measured for the freeze-thawed samples had to fall within 30% of the values measured for the untreated samples. For CMST and CMSTF/S, the average titers were 5.03 × 10^5^ PFU/mL (CV = 10.9%) and 4.44 × 10^5^ PFU/mL (CV = 21.3%), respectively, comprising only an 11.7% difference between the values for the two conditions (Figure S3). For CMPT and CMPTF/S, the average titers were 9.57 × 10^5^ PFU/mL (CV = 7.3%) and 1.01 × 10^6^ PFU/mL (CV = 12.2%), respectively, with a 5.8% difference between the values for the two conditions (Figure S3). For RMST and RMSTF/S, the average titers were 1.01 × 10^6^ PFU/mL (CV = 13.2%) and 8.63 × 10^5^ PFU/mL (CV = 9.3%), respectively, comprising only a 14.6% difference (Figure 4). For RMPT and RMPTF/S, the average titers were 1.48 × 10^6^ PFU/mL (CV = 19.0%) and 1.40 × 10^6^ PFU/mL (CV = 14.2%), respectively, with a 5.4% difference between the values (Figure 4). No statistically-significant difference (where *p* < 0.05) between titers was observed for the cynomolgus macaque samples nor the rhesus macaque plasma samples. A statistically-significant difference was observed between titers measured from the RMST and RMSTF/S sample set, where the *p*-value was calculated at <0.01. Based on the assay performance, the measured titers of RMST and RMSTF/S were not biologically different. They were within 30% of each other, even though mathematically, they are distinct datasets with low variation in the numbers. In total, these results indicated that it is not a detrimental practice to incur three freeze/thaw cycles on cell culture medium or nonhuman primate serum and plasma samples containing a filovirus concentration of about 10^3^ to 10^6^ PFU/mL during regular use.

#### 3.4.2. Change of Inoculum Volume and Tissue Culture Vessel

**Figure 5:**
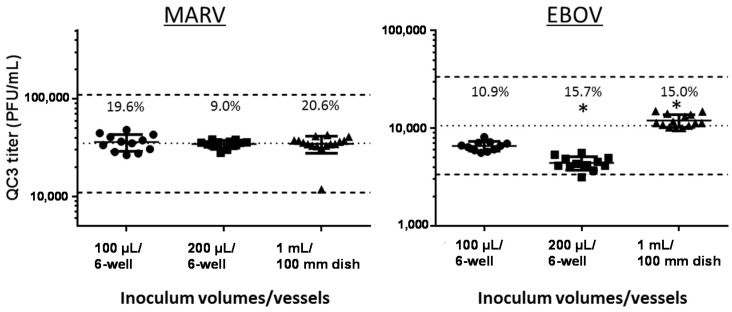
Various inoculum volumes and tissue culture vessels are suitable for use in the validated assay. The individual titers, averages, %CVs and standard deviations for *n* = up to 15 six-well plates or 100-mm dishes are presented for inoculum volumes of 100 µL (circles), 200 µL (squares) and 1 mL (triangles) for MARV and EBOV validation data. * Indicates statistical significance from the 100-µL group (Student’s *t*-test, *p* < 0.01). The central dotted line in the graphs represents the nominal titer for QC3 for MARV and EBOV, respectively. The dotted lines above and below the central line represent ±0.5 log_10_ for the nominal QC3 titer and the boundaries into which 80% of the titers must fall to pass the accuracy criterion in this assay. The QC3 titers that the dotted lines represent are presented in Table 1 and Table 2 for reference.

It is sometimes necessary to quantitate virus solutions with potentially very low titers using the plaque assay. Increasing the inoculum volume in the plaque assay to 200 µL or 1 mL lowers the lower limit of quantitation (LLOQ) to 50 PFU/mL or 10 PFU/mL, respectively, when plating undiluted samples and detecting a minimum of 10 plaques in the vessel. However, larger volumes need to be plated onto a sufficiently larger surface area of cells to maintain the right plating conditions for plaque formation. We previously evaluated the performance of various inoculum volumes and determined that larger volumes (e.g., >400 µL) plated onto a small area, e.g., a six-well plate with a surface area of only 9.5 cm^2^/well, may not promote adequate surface contact of virus particles to force them to be directed onto cell monolayers to make the optimal number of plaques [9]. The effect of inoculum volume variation was tested in EBOV and MARV validation studies by plating serial ten-fold dilutions of QC3 in 100-µL and 200-µL inoculum volumes on cells in six-well plates (growth area of 9.5 cm^2^/well) or 1-mL volumes on 100-mm dishes (55 cm^2^/well). For this experiment, one dilution series of QC3 was prepared in a volume sufficient to inoculate all vessels with the same dilution preparation at the same time. The test-specific acceptance criterion for this validation test was that 80% of the observed titers in each of the 100 µL, 200 µL and 1 mL sets must be within ± 0.5 log_10_ of the nominal titer for QC3 to demonstrate the accuracy of the method using the larger inoculum volume. All of these QC3 data points passed this criterion (Figure 5), and therefore, the use of 200 µL or 1 mL as the inoculum volume/vessel combination was an acceptable practice, resulting in accurate viral titration for EBOV and MARV, as compared to the other data collected in this validation, where 100 µL in a six-well plate was used most often. The observed values for the three conditions in the MARV validation were all very similar, with no statistical significance between the three datasets, as measured by Student’s *t*-test comparisons. The observed values were slightly different for the three conditions in the EBOV validation, but all of them fell within ± 0.5 log_10_ of the nominal titer for EBOV QC3. Statistical differences between the EBOV test groups were observed (Student’s *t*-test, *p* < 0.01), but because the titer values were still quite similar, there are no biologically-significant titer differences when these different inoculum volumes are plated in six-well plates or 100-mm dishes.

Evaluation of different volumes and larger culture dishes enabled examination of the LLOQ and the limit of detection (LOD) of filovirus plaques in this assay system. The LLOQ in this experiment depended on the vessel used, the inoculum volume plated and the observation of at least 10 plaques, as required by the plaque assay SOP. Using all of the raw plaque count data on the validation studies, it was observed that as few as 1, 2 or 3 plaques could be detected in wells or dishes at the lowest dilutions tested. It is important that an investigator be able to distinguish the presence of only one plaque compared to a monolayer blemish or other areas of damage. The negative control wells and dishes in these experiments were free of holes, blemishes or damage that could be confused for a singular plaque. The presence of singular or a small number of plaques at the lowest dilutions was believable due to the presence of increasingly higher numbers of plaques in the more concentrated dilutions in the series. For example, in dishes, the average EBOV plaque number in 1 mL of inoculum was 120 plaques at the 10^−2^ dilution, 12.3 at the 10^−3^ dilution and 2.92 at the 10^−4^ dilution. The 2.92 result was averaged from a series of 13 dishes with plaque numbers ranging from five to only one plaque per dish. The presence of so few true plaques was credible and supports an LOD of as little as one plaque, because it was in the context of more plaques seen at each of the next higher dilutions. However, using counts this low is not recommended for the calculation of an actual titer. The SOP supports counting plaques greater than 10 for assurance that a statistically-significant number of plaque events results in a robust quantifiable data result. For the LLOQ, 10 plaques in a 0.2-mL volume of undiluted sample, plated in a six-well plate, results in an LLOQ of 50 PFU/mL, whereas plating 1 mL of undiluted sample in a 100-mm culture dish results in an LLOQ of 10 PFU/mL. The LOD of merely 1 PFU/mL in the 100-mm dish is believable in the context of a dilution series. This validation supports an LOD of one plaque per unit volume plated, but investigators should strive to plate a dilution series to assure the presence of as many plaques as possible, especially when titrating a sample of unknown virus content.

#### 3.4.3. Optimal Day to Stain and Count Plaques

Building flexibility of staining and counting times into the validated assay is desirable for the ease of performance, provided that these practices do not incur unwanted variability. We previously reported that using a final concentration of 0.5% agarose and 4% to 5% (*v/v*) Gibco or Sigma neutral red vital dye in the secondary overlay produces optimal staining conditions [9]. In addition, we observed that the most optimal days to stain were Days 7 and 8 post-inoculation and reading within two days of staining [9]. These day-to-stain robustness tests were performed to determine if there was any difference in observed titers collected when plaques were stained on Day 7 and counted on Days 8 or 9, *versus* stained on Day 8 and counted on Days 9 or 10. Two sets of 75 plates each of Vero E6 cell plates were produced to perform both of these tests on the same day so that neither cell passage number nor day of performance could be considered variables; thus, all 150 plates were inoculated with the same virus preparations of PC, QC1, QC2 and QC3. One set of plates was stained on Day 7, for counting on Days 8 and 9, and one set was stained on Day 8, for counting on Days 9 and 10. The effect of altering staining day on calculated titer was evaluated through a comparison of titers at each of the days counted. The test-specific acceptance criterion for this assay stated if counting on the latter day produced results greater than ± 20% different than the earlier day, then counting on the earlier day will be chosen as the validated method. Percent variability was calculated as follows:

$$Variability\;\left(\%\right)=\;\frac{mean\;titer\;Day\;8\;-mean\;titer\;Day\;9}{\left(\left(mean\;titer\;Day\;8\;+mean\;titer\;Day\;9\right)\;÷2\right)}\;\times 100$$

Generally, many filovirus researchers with experience using the plaque assay accept that MARV and EBOV plaque assay plates should be stained on Day 7 or Day 8, and the plaques can be therefore counted on Day 8 or Day 9, respectively. The results from this well-controlled study generally agree with this practice. For most of the EBOV and MARV QC sample sets, there was less than 20% variability in the results when the same plates stained on Day 7 were counted on Day 8 or again on Day 9. The same was true for plates stained on Day 8 and counted on Day 9 or again on Day 10, but the Day 10 data collection time had limitations. Some EBOV and MARV QC datasets failed at the Day 10 collection time due to an increase in unreadable wells on the plates. More wells on Day 10 were Uncountable (U) due to total monolayer damage. These were the same monolayers that had looked good and were countable on Day 9 (Table 6). In general, the monolayers with stain on them for about 48 h looked worse and had more incidents of U scores than those that had been under stain for only about 24 h. Day 10 monolayers not only had been under stain for 48 h, but also were an additional day old, increasing the possibility of U monolayers. This phenomenon of an increase in U scores in all of the 144 wells on the second day was worse for MARV than EBOV, based on the data in Table 6, and especially for MARV QC1. It is difficult to state the reason for this observation, but it is possible that MARV plaques grow or spread faster and ruin the monolayer to the extent that it is uncountable by the second day under stain. Waiting until Day 10 to count plates can lead to data loss and would be a risky practice, and therefore, it is not recommended in the validated assay method.

**Figure 6:**
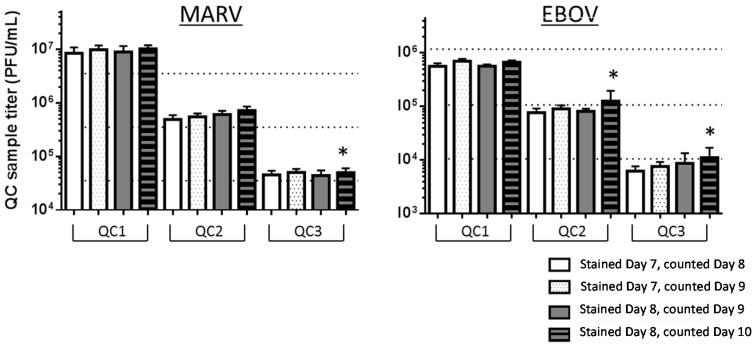
QC sample titers were acceptably similar if counted one or two days post-staining. The averages and standard deviations (*n* = 12) for QC1 to 3 titers stained and counted on Days 8, 9 or 10 are presented. The three horizontal dotted lines plot the nominal titers of MARV and EBOV QC1, QC2 and QC3 (Table 1 and Table 2). * Indicates a failed dataset, as described in Table 7.

The majority of the comparisons of titers counted at Day 8, 9 or 10 of the assay were within 20% of the first counted value (Figure 6 and Table 7). There was one instance in the EBOV data where the variability was calculated to be 22% higher on Day 9 than it was when counted on Day 8. However, this phenomenon of a >20% increase was not observed again with the QC2 and QC3 sets stained on Day 7 and counted on Day 8 *versus* again on Day 9. These % increases were 16.8% and 19.9%, respectively. If the practice of waiting one extra day to count the plaques were truly introducing variability in the titer over the original value collected at the first day after staining, it is hypothesized that this observation would have been more robust, and it would have been noted for all three of the QC sets, no matter what their nominal titer. There were no instances in the MARV dataset where the titer was greater than 20% when counted on the latter day. Moreover, the test results for all conditions fell within the accuracy criteria for each QC set, meaning that the titers measured for each QC set were within ±0.5 log of the nominal titer for each QC set. The assay is valid for plates stained on Day 7 and counted on Days 8 and 9 or stained on Day 8 and counted on Day 9.

#### 3.4.4. Cell Seeding and Time of Cell Use

Validation tests were performed to evaluate the performance of cells at a particular concentration and passage number when used at 24, 48 or 72 h after seeding. These experiments were done to test assay flexibility. Building in the option to use cells one day after the planned usage could ease research performance in unexpected circumstances where the need for the plaque assay performance becomes delayed by one day (e.g., delay due to inclement weather or illness). Plated cells represent time, effort and money, which get wasted if they cannot be used, and replating could delay a study by a week or more.

**Figure 7:**
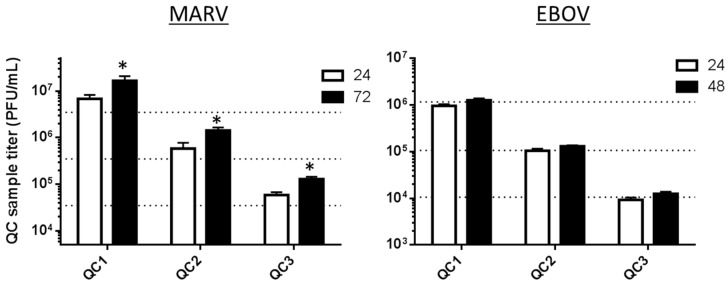
Use of cells at 24 or 48 h post-seeding provides accurate titer data for the EBOV validated plaque assay, but use at 72 h fails in the MARV assay. The bars represent average measured titers for QC1, QC2 or QC3 for MARV or EBOV, measured on cells plated at 24, 48 or 72 h prior to infection. The three horizontal dotted lines plot the nominal titers of MARV and EBOV QC1, QC2 and QC3. Those nominal titers are presented in Table 1 and Table 2. The * indicates failed runs for MARV tested on cells plated 72 h before use.

Tests on the EBOV validation were performed to evaluate the performance of cells at a particular concentration and passage number (e.g., Passage 33) used 24 ± 6 h after seeding or used 48 ± 6 h after seeding. The concentration of cells plated was 700,000 ± 25,000 cells/well. The MARV validation experiment, which was completed before the EBOV validation was undertaken, differed slightly in that the cell plating ages tested were 24 and 72 ± 6 h after seeding, but the 48-h range was not tested. For both validations, the first batch of cells was observed at 24 h post-seeding to be 96% to 98% confluent and was used at that time. The second batch was observed at 48 (or 72 for MARV) hours post-seeding, and these were found to be heavily confluent (100%) and to appear tightly packed when they were used, especially for the 72-h-old cells. The MARV and EBOV assays evaluated the titers of the PC and QC1 to QC3 on each of these cell ages. The results for the 24-h and 48-h-old cells tested on the EBOV validation passed all assay performance acceptance criteria (Figure 7). The titers for EBOV PC and QC1 to QC3 were all acceptably within the nominal titer for each QC set, whether they were measured on 24-h-old cells or 48-h-old cells. The titers for MARV PC and QC1 to QC3 measured on 24-h-old cells were all acceptably within the nominal titer for each QC set, but the 72-h-old cells did not produce passing results for the PC or any of QC1 to 3 because all of the titers were too high to fall within ±0.5 log_10_ of the nominal titers of those samples.

It is possible that the performance of the MARV assay on 48-h-old cells in a validation bridging experiment would pass, and this is a future direction which will be evaluated to expand the MARV validation package. Another concept that should be tested in future validation bridging experiments for MARV and EBOV would be to plate cells at lower concentrations (e.g., 300,000 cells/well), evaluating by microscopic observation whether they can achieve adequate confluency by 72 h and determining if passing titer results can be obtained. Successful results in an experiment designed in this manner would help build flexibility into the performance schedule for assays on a weekly cadence. Currently, assays must be performed based on the cell seeding schedule in the USAMRIID cell culture lab, limiting the days of the week on which GLP-compliant assays may be performed.

#### 3.4.5. Cell Passage Number

As presented in a previous filovirus plaque assay standardization report [9] and promoted as an ideal standardized reagent [20], the BEI Vero E6 cells are the Filovirus Animal Nonclinical Group’s (FANG) cell line of choice for the performance of the standardized filovirus plaque assay. Thus, this cell line was chosen for the validation activities for MARV and EBOV. Tests on the MARV validation were performed with Vero E6 cells from BEI (C1008) Catalog No. NR-596, but they were Lot Number 3956812, which has not been as well characterized as the now-preferred Lot Number 3956593. The Lot 3956812 cell passage ages tested in the MARV validation experiments ranged from 21 to 49, and those results agree closely with published data, indicating that any passage age in that range produced passing titers for the MARV PC and QC1 to 3 [9]. A bridging MARV validation experiment with the preferred lot of cells was completed and indicated that the new lot, grown under GLP-compliant conditions at USAMRIID, functioned similarly at the passage age of 31, with older passages to be tested in the future. The EBOV validation experiments began using Vero E6 cells from BEI Lot 3956812, and tested Passages 32 to 36.

**Figure 8:**
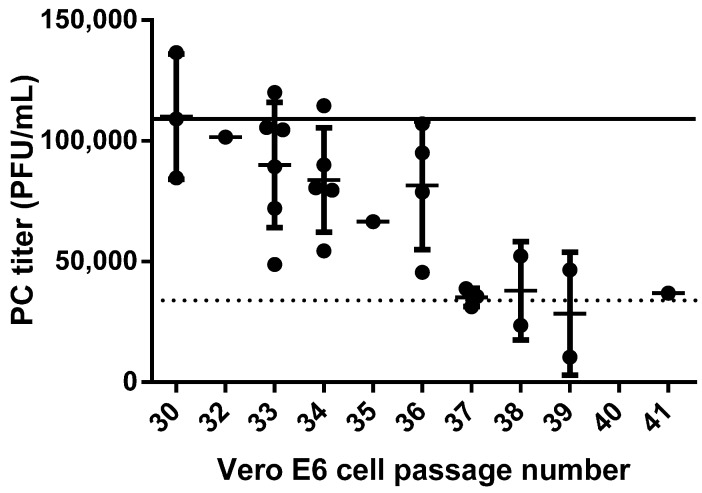
Positive control data collected on Vero E6 cells of various passage ages. Twenty eight measurements of the EBOV Positive Control (PC) collected over pre-, post- and on-validation studies were performed on cell passages ranging from 30 to 41 (filled circles). Averages and standard deviations are presented for multiple results on the same passage. The solid line depicts the nominal PC target titer of 1.09 × 10^5^ PFU/mL, and the dotted line denotes the lower PC cutoff limit of 34,469 PFU/mL, below which the assay fails and a repeat is required.

Tracking the performance of the PC on a variable such as cell passage number comprises a system suitability evaluation for the validation. System suitability helps the user evaluate the performance of the assay over time, and if a factor like the PC titer begins to fail routinely, then it can be an indicator that something in the system (e.g., the cell passage number or a similar assay constituent) is no longer providing a consistent assay system. The value for the EBOV PC in this validation had to fall within ±0.5 log_10_ of the nominal titer of the PC, or the performance of the assay was deemed as a failure. The range in which the PC titer was required to fall was 34,469 to 344,688 PFU/mL, with a nominal target titer of 1.09 × 10^5^. The PC titer was recorded for experiments performed pre-validation, during the GLP validation and peripheral to the validation in well-documented research studies and is presented *vs.* passage number in Figure 8. It was observed that the PC titer was reduced as the cell passage age increased. All other assay constituents, such as medium, FBS, agarose, cell culturing practices, cell concentration, *etc.*, had all remained constant, as recorded in the GLP-compliant study records.

The conclusions from this compilation of PC data support the use of cells at only Passage 36 and younger for the validated EBOV assay. The cutoff of 36 was chosen due to the number of data points which fell comfortably above the PC cutoff limit, unlike those for Passages 37 to 40, even though some of those values passed. The limitation of cell culture passage age 36 as the cutoff for EBOV plaque assay methods may be in contrast with the findings for the validated MARV assay. For the MARV assay, the other lot of Vero E6 cells was evaluated and was not found to have a passage age limitation [9]. Cells at passage ages younger than 31 or 32 are generally not available from USAMRIID cell culture due to the laboratory’s receipt of the cell stock at Passage Age 24 from BEI Resources. Once the cells were received, they were amplified several passages and banked for frozen storage. Banked frozen cells are thawed and passaged in culture to fill orders, thus an order for six-well plates can typically be filled with cells only as young as Passage 31 or 32. Acceptance of the narrow window of cell passage ages affects the GLP-compliant USAMRIID cell culture lab and dictates the ages of cells they may maintain in passage for filling orders for validated studies.

## 4. Discussion

Different filovirus challenge stocks can have different provenances, which may impact their ability to produce equivalent-looking plaques in a plaque assay. The studies in this EBOV validation were completed with a virus preparation of EBOV/Kikwit, which has been in use for MCM evaluations at USAMRIID [21,22]. The stock was produced in cell culture (Passage 3) and is known to have a predominantly 8U genomic phenotype [1]. When grown *in vitro* (e.g., Vero E6 cells or similar), EBOV appears to have a preferred 8U genomic state, but a preferred 7U genomic state *in vivo* [1,23]. Hence, a 7U mRNA editing site is perhaps required for viral fitness in a mammalian host, where it apparently makes and secretes a higher amount of sGP. Conversely, an 8U genomic phenotype may have a growth advantage in culture. It could enhance virus particle release *in vitro*, making it the genomic phenotype responsible for high titer growth of virus stocks *in vitro* [23,24]. Use of either the 8U or the 7U genotypes may be preferred as a virus challenge stock in NHP studies in some filovirus research circles [24,25]. Use of either the 8U or the 7U variants may be justifiable for animal-rule studies to develop the NHP infection model itself or to test the efficacy of vaccines or therapeutics [20]. Beyond the preparations of EBOV-Kikwit, which are distinguishable as 7U or 8U, the EBOV-Makona variant is also now being amplified as a challenge stock reagent for animal studies, with the intent of investigating the EVD outbreak caused by this variant in 2013 to 2016 [26,27]. The MARV Angola variant is in heavy use for MARV animal rule studies supporting MCM countermeasure development [20], but other variants of MARV, such as Hesse (Musoke and Ci67 isolates), could also be used for important research [28,29].

The existence of these multiple variants of virus prepared as useful stocks, and the implications each brings, has a bearing on the validated filovirus plaque assay. The EBOV validation was completed with EBOV-Kikwit, a lot that may have the highest level of cell culture adaptation, if indeed the 8U genomic phenotype is a marker of *in vitro* production. This virus preparation, or lot, was used to prepare the PC and QCs, and they served as the working test samples in the EBOV validation assay. The assay was validated based on the behavior of these PC and QC samples against reasonable acceptance criteria set a posteriori from optimization and characterization study data. If a different lot of virus, comprised of a different variant or genomic phenotype, is tested in either the MARV or EBOV validated assay, it may or may not perform equivalently well in the assay validated for the original test variant or lot. The United States Food and Drug Administration (FDA) guidance for biological assay validations indicates that any change to critical reagents, such as the PC, or test samples, or the species of a matrix, will likely require a partial validation [7]. A partial validation can bridge the performance of the new variable to the performance of the old in the known validated method. Additional virus variants, stocks or lot preparations, cell lines or other major variables will need to be assessed for any potential changes that they may bring to the validated assay, and new PCs or QCs may be required for full testing. For example, the “7U” preparation lot or Makona variant may have a completely different plaque appearance than the previously-validated variant. Bridging validation studies may be needed to ascertain whether the hypothetical new plaque appearance can be similarly quantitated independently by two analysts in detailed precision studies, such as the ones presented in this work. Alternatively, plaques from an untested lot may need more or less time to develop into the most robust and countable plaques possible. Robustness and optimization studies may be required before such a bridging validation, to demonstrate that a certain day is superior for counting. No matter the genotype or provenance, it will be important to test new virus lots in characterization and optimization studies, before assuming the current validated assay will perform optimally for the new lot.

## 5. Conclusions

A filovirus plaque assay has been standardized and validated using GLP-compliant methods, proving that the assay is suitable for reliable and reproducible measurement of the MARV-Angola and EBOV-Kikwit virus variants in stock solutions, animal serum and plasma samples. The validation parameters tested included accuracy, precision, linearity, robustness, stability of the virus stocks and system suitability. Inter- and intra-assay variation were tested and results indicated a high level of agreement when plaques were counted and the titers were calculated by different analysts, demonstrating inherent consistency in plaque observations between trained technicians. Continuous work on the assay will be required to maintain the assay’s performance, ensuring no changes or drift in the performance of the critical reagents. While the assay has been validated for the parameters tested, future bridging studies to create new critical reagents (such as new PC and QCs for new variants), and/or validate new parameters or tissue matrices, will be required to ensure the assay evolves with the continuing needs of the research programs and can be proven to be fit for its intended purpose. 

## Figures and Tables

**Table 1:** Ebola Virus (EBOV) accuracy results in the context of the ranges of acceptable low and high % differences for both sets of Quality Control 1 (QC1), QC2 and QC3.

Sample	Nominal Titer ^1^	Low End of Range (PFU/mL) (this number is −0.5 log_10_)	High End of Range (PFU/mL) (this number is +0.5 log_10_)	% Difference of Low End from Nominal Titer	% Difference of High End from Nominal Titer
QC1	1.17 × 10^6^	3.70 × 10^5^	3.70 × 10^6^	−68.4%	+216%
EBOV QC1 average observed titer (*n* = 12) was 6.18 × 10^5^ PFU/mL (CV = 3.9%). This is −47.2% different from nominal, and the value passes.					
QC2	1.07 × 10^5^	3.38 × 10^4^	3.38 × 10^5^	−68.4%	+216%
EBOV QC2 average observed titer (*n* = 12) was 6.99 × 10^4^ PFU/mL (CV = 16.2%). This is a −34.7% difference from nominal, and the value passes.					
QC3	1.06 × 10^4^	3.35 × 10^3^	3.35 × 10^4^	−68.4%	+216%
EBOV QC3 average observed titer (*n* = 12) was 5.86 × 10^3^ PFU/mL (CV = 11.0%). This is a −44.7% difference from nominal, and the value passes.					

^1^ Nominal titers for EBOV QC1 to QC3 were determined just prior to beginning the validation by the measurement of these prepared QC stocks using the EBOV plaque assay method [9].

**Table 2:** Marburg Virus (MARV) accuracy results in the context of the ranges of acceptable low and high % differences for both sets of QC1, QC2 and QC3.

Sample	Nominal Titer ^1^	Low End of Range (PFU/mL) (this number is −0.5 log_10_)	High End of Range (PFU/mL) (this number is +0.5 log_10_)	% Difference of Low End from Nominal Titer	% Difference of High End from Nominal Titer
QC1	3.5 × 10^6^	1.096 × 10^6^	1.096 × 10^7^	−68.7%	+213%
MARV QC1 average observed titer (*n* = 12) was 2.88 × 10^6^ PFU/mL (CV = 34.9%). This is −17.7% different from nominal, and the value passes.					
QC2	3.5 × 10^5^	1.096 × 10^5^	1.096 × 10^6^	−68.7%	+213%
MARV QC2 average observed titer (*n* = 12) was 3.27 × 10^5^ PFU/mL (CV = 23.6%). This is a -6.4% difference from nominal, and the value passes.					
QC3	3.5 × 10^4^	1.096 × 10^4^	1.096 × 10^5^	−68.7%	+213%
MARV QC3 average observed titer (*n* = 12) was 4.95 × 10^4^ PFU/mL (CV = 20.1%). This is a 41.4% difference from nominal, and the value passes.					

^1^ Nominal titers for MARV QC1 to QC3 were based on the original titer of the MARV stock (3.5 × 10^7^ PFU/mL) diluted 1:10, 1:100 and 1:1000, respectively, to make the three QC preparations.

**Table 3:** Tests on the validation, which were of a similar design to the accuracy tests and passed the accuracy criteria.

Test	Number of EBOV Assay Runs within Nominal Titer Range for Each QC (% Difference of Average Observed Titer from Nominal)	Number of MARV Assay Runs within Nominal Titer Range for Each QC (% Difference of Average Observed Titer from Nominal)
Precision 1	QC1 12 of 12 (−18.1%)	QC1 12 of 12 (86.2%)
	QC2 11 of 12 (6.3%)	QC2 12 of 12 (105.8%)
	QC3 12 of 12 (3.3%)	QC3 10 of 12 (175.7%)
Precision 2	QC1 12 of 12 (105.5%)	QC1 12 of 12 (98.8%)
	QC2 12 of 12 (111.4%)	QC2 12 of 12 (89.4%)
	QC3 12 of 12 (−10.5%)	QC3 12 of 12 (90.1%)
Precision 3	QC1 11 of 12 (−52.1%)	QC1 12 of 12 (29.2%)
	QC2 12 of 12 (−30.7%)	QC2 12 of 12 (37.6%)
	QC3 12 of 12 (−43.9%)	QC3 12 of 12 (84.9%)
Precision 4	QC1 12 of 12 (−43.9%)	QC1 12 of 12 (30.6%)
	QC2 12 of 12 (−42.7%)	QC2 12 of 12 (14.8%)
	QC3 11 of 12 (−48.1%)	QC3 11 of 12 (36.3%)
Change of inoculum volume	QC1 N/A due to experimental design	QC1 N/A due to experimental design
	QC2 N/A due to experimental design	QC2 N/A due to experimental design
	QC3 12 of 12 (−38.3%)	QC3 12 of 12 (2.9%)
Stability	QC1 12 of 12 (−54.3%)	QC1 12 of 12 (109.9%)
	QC2 12 of 12 (−50.8%)	QC2 12 of 12 (83.0%)
	QC3 11 of 12 (−48.1%)	QC3 11 of 12 (95.4%)
Cell Seeding Time (24 h)	QC1 12 of 12 (−17.3%)	QC1 12 of 12 (94.3%)
	QC2 12 of 12 (−2.1%)	QC2 12 of 12 (67.0%)
	QC3 12 of 12 (−12.0%)	QC3 12 of 12 (68.1%)
Cell Seeding Time (48 h)	QC1 12 of 12 (9.0%)	Not performed *
	QC2 12 of 12 (22.0%)	
	QC3 12 of 12 (18.4%)	
Day to Stain	QC1 12 of 12 (−52.4%)	QC1 12 of 12 (141.6%)
	QC2 12 of 12 (−28.5%)	QC2 12 of 12 (39.9%)
	QC3 12 of 12 (−40.0%)	QC3 12 of 12 (29.2%)

* The MARV validation did not include an examination to determine if cells plated at 700,000 ± 25,000 cells/well could be used successfully at 48 h post-seeding.

**Table 4:** Variability in counts measured by two analysts is minimal for the EBOV and MARV plaque assays.

Test Counted by both Analysts	% Variability in EBOV Plaque Counts between Two Analysts			% Variability in MARV Plaque Counts between Two Analysts		
	QC1	QC2	QC3	QC1	QC2	QC3
Test 1 (run by Analyst A)	0.6%	2.9%	9.3%	17.8%	15.7%	13.5%
Test 2 (run by Analyst B)	6.0%	3.4%	1.9%	28.2%	27.4%	26.8%
Test 3 (run by Analyst A)	6.5%	7.2%	4.2%	9.5%	7.3%	4.8%
Test 4 (run by Analyst B)	3.6%	6.5%	1.3%	15.1%	8.3%	8.6%

**Table 5:** Intra-Class Correlation (ICC) analysis reveals a high level of agreement in plaque counts between two analysts collecting data using the same methods.

EBOV				MARV			
Precision Test/Analyst	Intra-class Correlation	95% Confidence Limits		Precision Test/Analyst	Intra-class Correlation	95% Confidence Limits	
		Lower	Upper			Lower	Upper
Test 1	0.99013	0.98809	0.99182	Test 1	0.92346	0.90471	0.93819
Test 2	0.99490	0.99384	0.99578	Test 2	0.92959	0.89137	0.95150
Test 3	0.96788	0.96130	0.97334	Test 3	0.96623	0.95935	0.97197
Test 4	0.96507	0.95796	0.97100	Test 4	0.96050	0.95105	0.96798

**Table 6:** Day 10 monolayers become fragile and deteriorate, increasing the number of uncountable (U) wells across all dilutions plated in the assay.

# U Wells in 144 Wells Plated (% of Total Wells)	QC1	QC2	QC3
Stained Day 7, counted Day 8	5 (3.5%)	0 (0%)	1 (0.7%)
Stained Day 7, counted Day 9	4 (2.8%)	0 (0%)	2 (1.4%)
Stained Day 8, counted Day 9	6 (4.2%)	0 (0%)	2 (1.4%)
Stained Day 8, counted Day 10	66 (45.8%)	79 (54.9%) *	56 (38.9%) *
**# U Wells in 144 Wells Plated (% of Total Wells)**	**QC1**	**QC2**	**QC3**
Stained Day 7, counted Day 8	24 (16.7%)	1 (0.7%)	4 (2.8%)
Stained Day 7, counted Day 9	67 (46.5%)	5 (3.5%)	4 (2.8%)
Stained Day 8, counted Day 9	58 (40.3%)	1 (0.7%)	23 (16.0%)
Stained Day 8, counted Day 10	96 (66.7%)	56 (38.9%)	41 (28.5%) *

* Uncountable (U) wells in the best countable dilutions on the plates were so numerous that the indicated QC set failed to provide the minimum number of data points to satisfy the acceptance criteria for successful day-to-stain assay performance; # = number.

**Table 7:** Counting on one and two days following staining is an acceptable practice for the validated plaque assay.

Virus	First Plate Set		Second Plate Set	
	Counted Day 8 (Stained Day 7)	Counted Day 9 (Stained Day 7)	Counted Day 9 (Stained Day 8)	Counted Day 10 (Stained Day 8)
**EBOV**	QC1 = 5.57 × 10^5^	QC1 = 6.95 × 10^5^ 22% titer increase	QC1 = 5.63 × 10^5^	QC1 = 6.71 × 10^5^ 17.5% titer increase
	QC2 = 7.65 × 10^4^	QC2 = 9.05 × 10^4^ 16.8% titer increase	QC2 = 8.13 × 10^4^	QC2 = dataset failed *
	QC3 = 6.25 × 10^3^	QC3 = 7.63 × 10^3^ 19.9% titer increase	QC3 = 8.74 × 10^3^	QC3 = dataset failed *
**MARV**	QC1 = 8.46 × 10^6^	QC1 = 9.79 × 10^6^ 14.5% titer increase	QC1 = 8.95 × 10^6^	QC1 = 1.02 × 10^7^ 13.2% titer increase
	QC2 = 4.90 × 10^5^	QC2 = 5.50 × 10^5^ 11.5% titer increase	QC2 = 6.06 × 10^5^	QC2 = 7.22 × 10^5^ 17.5% titer increase
	QC3 = 4.52 × 10^4^	QC3 = 5.02 × 10^4^ 10.4% titer increase	QC3 = 4.40 × 10^4^	QC3 = dataset failed *

* Datasets failed due to the presence of too many unscorable wells and too few counts resulting from the assay set to meet the assay performance acceptance criteria.

## Supplementary Materials

The following are available online at http://www.mdpi.com/1999-4915/8/4/113/s1. **Figure S1**. Bland–Altman analysis demonstrates a visual representation of the agreement between plaque numbers counted by two individual analysts. **Figure S2**. Freeze-thaw treated (designated as F/S) and untreated quality control QC samples 1-3 have very similar measured titers in the MARV plaque assay. **Figure S3.** MARV-spiked cynomolgus macaque serum and plasma can be titered after freeze-thaw cycles. Cynomolgus Macaque Serum (CMST) and Plasma (CMPT) were subjected to three freeze-thaw cycles of being held thawed at ambient temperature for at least 30 min and refrozen at −60 °C or colder for no less than 20 h, on three consecutive days. Samples were titered in the plaque assay after the fourth thaw.
