# Supplementary material for: Validation of the Filovirus Plaque Assay for Use in Preclinical Studies
Source: Viruses. 2016 Apr 21;8(4):113. doi: 10.3390/v8040113 (PMC4848606; doi:10.3390/v8040113)
Supplement: Supplementary file 1 [file viruses-08-00113-s001.pdf]

Supplementary Materials: Validation of the Filovirus Plaque Assay for Use in Preclinical Studies

Amy C. Shurtleff, Holly A. Bloomfield, Shannon Mort, Steven A. Orr, Brian Audet, Thomas Whitaker, Michelle J. Richards and Sina Bavari

The Bland-Altman plot below is a visual representation of the agreement of the MARV plaque counts made by the two analysts for each Precision experiment. The difference between counts of each well are plotted on the y-axis, while the mean of the two counts for each well are plotted on the x-axis. The central reference line in each plot is the mean of the differences between counts; while the two other reference lines represent two standard deviations of the differences above and below the mean. It appears that the fewer the plaques counted, the closer the data fell to that central reference line: therefore, it is possible that when there are fewer plaques to count, there is less variation between two analysts. The Bland-Altman analysis results for MARV are very similar to those seen for the EBOV analysis.

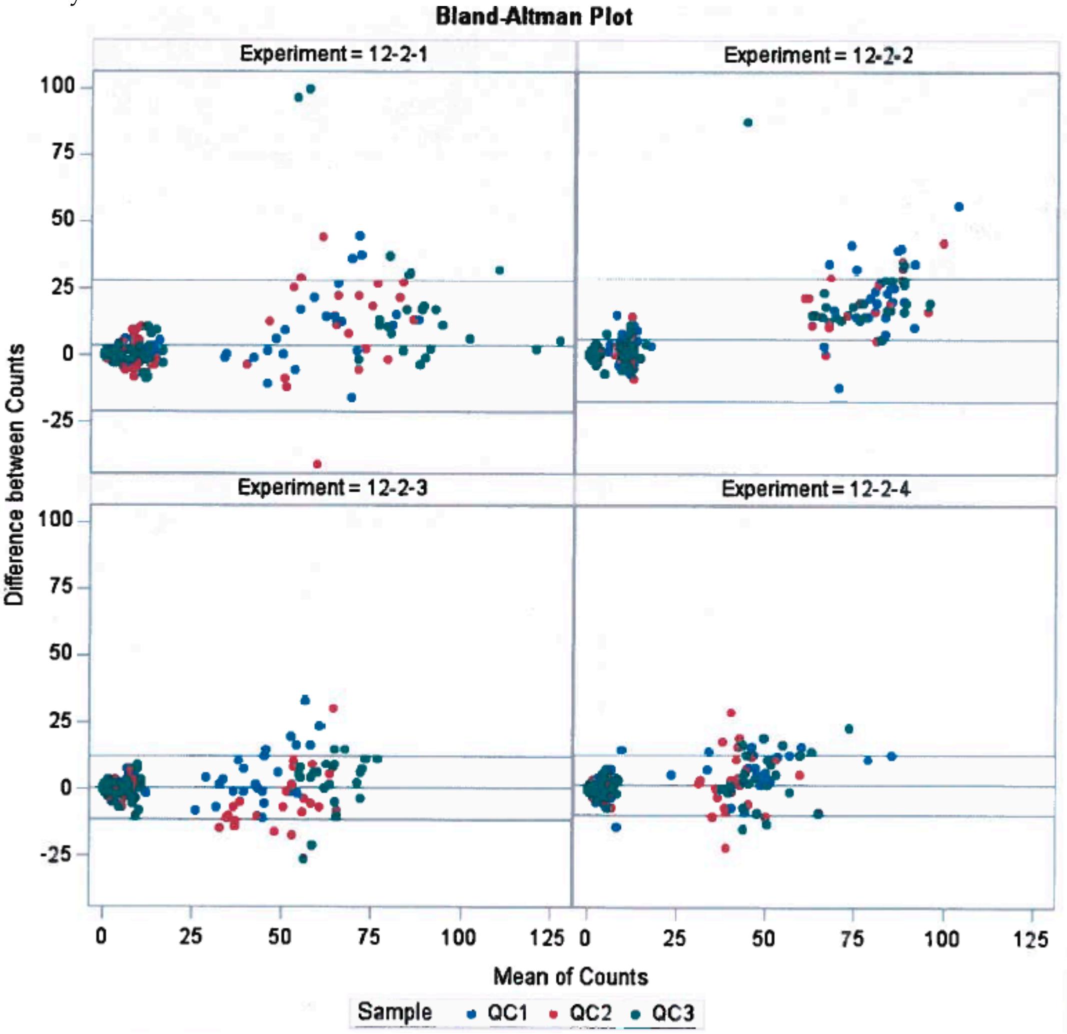

Figure S1. Bland–Altman analysis demonstrates a visual representation of the agreement between plaque numbers counted by two individual analysts.

The validation protocol acceptance criterion for stability was that the F/S sample had to return a titer no more than 30% different from the value measured for the non-freeze-thawed sample to demonstrate that freeze-thawing had no detrimental effect on the sample. Consistently, less than a 30% difference was observed for the MARV cell culture samples, where the widest difference from the untreated sample was 12.4% for MARV QC1 (see below). Statistical comparison by Student's *t*-test of the treated *versus* untreated samples demonstrated no statistical significance between any of

the treated or untreated datasets. The practice of using a cell culture sample that has up to three freeze-thaw manipulations is acceptable for the EBOV and MARV validated assays.

QC and QCF/S set	% Difference from Value Measured for Untreated QC Sample	Student's *t*-test
QC1 and QC1F/S	12.4	$p = 0.13$
QC2 and QC2F/S	8.8	$p = 0.38$
QC3 and QC3F/S	−1.6	$p = 0.86$

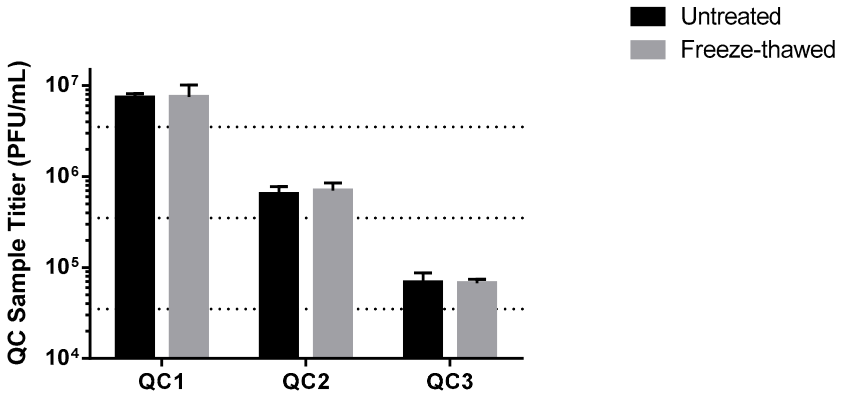

Figure S2. Freeze-thaw treated (designated as F/S) and untreated quality control QC samples 1-3 have very similar measured titers in the MARV plaque assay.

MARV-spiked cynomolgus macaque serum and plasma can be titered after freeze-thaw cycles.

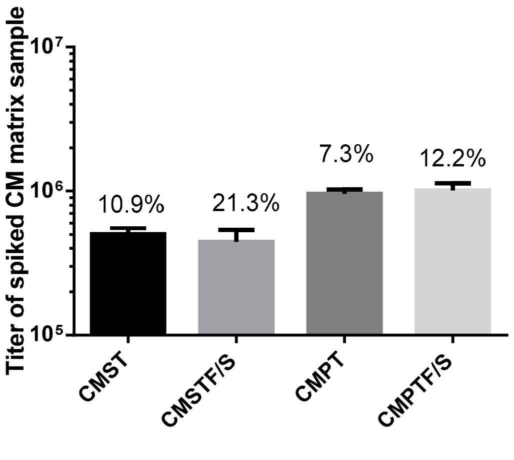

Figure S3. MARV-spiked cynomolgus macaque serum and plasma can be titered after freeze-thaw cycles. Cynomolgus Macaque Serum (CMST) and Plasma (CMPT) were subjected to three freeze-thaw cycles of being held thawed at ambient temperature for at least 30 min and refrozen at −60 °C or colder for no less than 20 h, on three consecutive days. Samples were titered in the plaque assay after the fourth thaw. There was little variability in each average titer, and a Student's *t*-test found a statistically-significant difference ($p < 0.01$) between CMST and CMSTF/S sample sets, yet the titers are acceptably similar; therefore, the difference is not biologically significant.

© 2016 by the authors; licensee MDPI, Basel, Switzerland. This article is an open access article distributed under the terms and conditions of the Creative Commons by Attribution (CC-BY) license (http://creativecommons.org/licenses/by/4.0/).
